# Finding an Optimal Level of GDNF Overexpression: Insights from Dopamine Cycling

**DOI:** 10.1007/s10571-023-01375-z

**Published:** 2023-07-06

**Authors:** Pepin Marshall

**Affiliations:** 1 Neuroscience Center, University of Helsinki, 00014 Helsinki, Finland; 2 Institute of Pharmacology, Toxicology and Pharmacy, Ludwig-Maximilians-University, Munich, Germany

**Keywords:** GDNF, Parkinson’s, Dopamine, DAT, Treatment, Hyperdopaminergia

## Abstract

The application of glial cell line-derive neurotrophic factor (GDNF) to cell cultures and animal models has demonstrated positive effects upon dopaminergic neuronal survival and development, function, restoration, and protection. On this basis, recombinant GDNF protein has been trialled in the treatment of late-stage human Parkinson’s disease patients with only limited success that is likely due to a lack of viable receptor targets in an advanced state of neurodegeneration. The latest research points to more refined approaches of modulating GDNF signalling and an optimal quantity and spatial regulation of GDNF can be extrapolated using regulation of dopamine as a proxy measure. The basic research literature on dopaminergic effects of GDNF in animal models is reviewed, concluding that a twofold increase in natively expressing cells increases dopamine turnover and maximises neuroprotective and beneficial motor effects whilst minimising hyperdopaminergia and other side-effects. Methodological considerations for measurement of dopamine levels and neuroanatomical distinctions are made between populations of dopamine neurons and their respective effects upon movement and behaviour that will inform future research into this still-relevant growth factor.

**Graphical Abstract:**

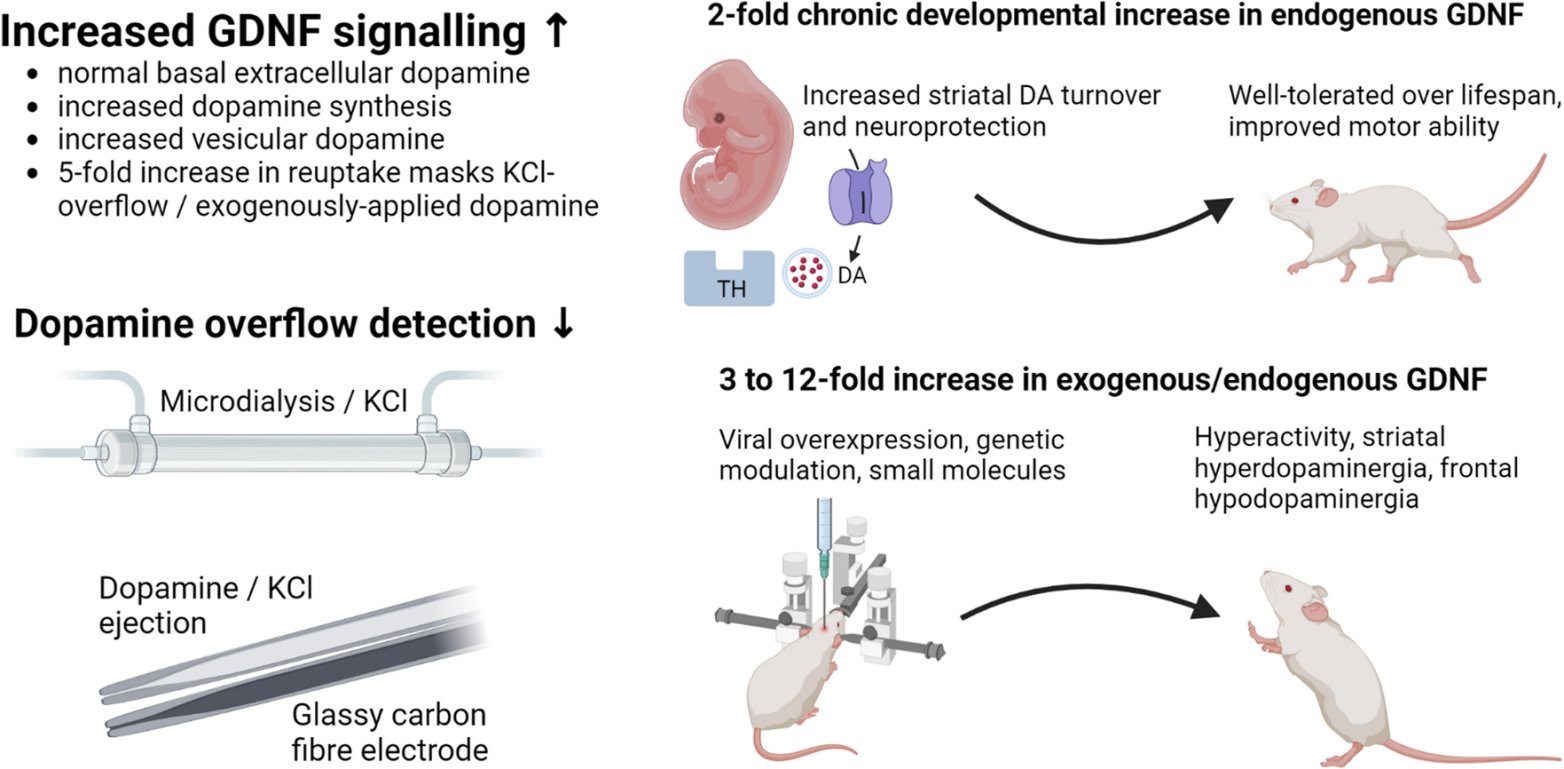

## Introduction

The discovery of glial cell line-derived neurotrophic factor (GDNF) as a secretion product from the rat B49 glioma cell line in 1993 heralded the arrival of a powerful promotor of dopaminergic cell survival with enormous potential for treating Parkinson’s disease (PD). Lin et al (1993) demonstrated greatly increased differentiation, survival, and neurite outgrowth of embryonic nigral dopaminergic neurons in culture and increased dopamine reuptake—likely as a result of increased tyrosine hydroxylase-positive neuronal body size and neurite outgrowth. These dopaminotrophic effects are achieved by GDNF signalling through the receptor tryosine kinase known as RET (rearranged during transfection; Durbec et al. 1996) after binding to a glycosyl-phosphatidylinositol (GPI)-linked co-receptor known as GFRα1 (Jing et al. 1996; Treanor et al. 1996). Upon binding, RET dimerises and activates intracellular cascades, the activation or inactivation of which have broad effects upon the development of cancers, endocrine neoplasias, peripheral nerves, spermato- and nephrogenesis, and upon dopamine neuron development and maintenance. The GFRα1-RET receptor complex is expressed by all of the pacemaking A9 dopaminergic neurons of the substantia nigra pars compacta (SNpc) and is necessary for the survival-promoting effects of GDNF upon dopaminergic neurons (Durbec et al. 1996; Treanor et al. 1996; Drinkut et al. 2016; Mahato et al. 2020). See Kramer and Liss (2015) and Conway et al. (2020) for comprehensive reviews. There is a wealth of evidence that GDNF can protect, restore, and augment dopaminergic function in the nigrostriatal pathway in acute animal models of Parkinson’s disease (Conway & Kramer 2022). Interestingly, GDNF seems to be of particular importance during development, since notably higher levels of GDNF mRNA have been detected in the developing compared to the adult striatum [N.B. these are rat studies (Schaar et al. 1993; Strömberg et al. 1993)]. GDNF is still expressed in low concentrations in the adult striatum and retrogradely tranpsorted to nigral dopaminergic neurons, suggesting a trophic role in the adult (Tomac et al. 1995; Barroso-Chinea et al. 2005). Despite success in acute animal models of PD, clinical trials in PD patients using intracranial injection of GDNF have shown limited positive effects. This may be due to a lack of the nigrostriatal axonal terminals required to retrogradely transport GDNF back into the pacemaking cells of the substantia nigra and, coupled with poor diffusibility of GDNF in the striatum in its natively expressed form, may explain the current lack of a viable GDNF therapy (Manfredsson et al. 2020). Earlier treatment and more easily diffusible GDNF variants, as well as small molecule RET agonists and methods of endogenous upregulation of GDNF are currently being explored. 

Side- and off-target effects are important to consider for future therapies as they affect the tolerability and utility of GDNF. Side-effects following intracerebroventricular (icv) GDNF injection in humans are documented as principally nausea, vomiting, paresthesias, hyponatremia, anorexia, and weight loss (see Barker et al. 2020) and cerebellar toxicity at high doses in macaques (Luz et al. 2016), whilst intraputamenal injection is better-tolerated yet results in electric dysesthesias (Lhermitte’s phenomenon). These side-effects are hypothesised to be due to the wide variety of off-target receptors and diffusion of GDNF into the cerebrospinal fluid. More refined approaches to increase endogenous GDNF in a cell-specific manner, or to modulate specific receptor complexes may overcome these effects. They may also produce more dopamine-centric psychiatric side-effects due to the strongly enhancing effects of GDNF upon dopamine turnover. However, whilst the broader effects of GDNF upon dopamine signalling and behaviour are documented in the literature on animal models they have not been comprehensively summarised and are seen as “difficult to interpret” (Mätlik et al. 2022).

The aim of this review is to evaluate the effects of increasing GDNF levels or RET/GDNF signalling in healthy nervous tissue in adult animals, cell cultures, and acute brain preparations, either exogenously via direct application or viral transduction, or endogenously via native upregulation. Papers that contained at least one of these themes were selected for inclusion; see Table 1 for summary.

**Table 1:** Inclusion criteria for review; papers must contain at least one dopaminergic measure in response to altered GDNF/RET signalling

Effect of GDNF/RET upon	Measured via
Dopamine production and storage	Dopamine levels in tissue, in vivo microdialysis, electrochemistry
Dopamine cycling	Metabolite levels from tissue, in vivo microdialysis
Dopamine release	In vivo microdialysis, in vivo/in vitro electrochemistry in response to chemical/electrical stimulation
Dopamine reuptake kinetics	Radiolabelled dopamine/electrochemistry in vivo/in vitro
Dopaminergic cell morphology	Size of soma, branching complexity, synaptic puncta
Dopaminergic cell marker expression	Immunohistochemistry or Western blot for tyrosine hydroxylase (TH), dopamine transporter (DAT), vesicular monoamine transporter (VMAT2)
Electrophysiological function	In vivo microarray, in vitro patch-clamp
Motor function	Coordination, balance, grip strength, motor learning
Behaviour	Pre-pulse inhibition, feeding, hyperactivity, lethargy, anxiety, depression, sociability

## GDNF Promotes Dopaminergic Phenotype, Dopamine Turnover, and is Excitatory in Cultured Midbrains Cells

The first published literature on GDNF showed dopamine uptake increased 2.5 to threefold per cultured midbrain neuron (Lin et al. 1993) in concert with a qualitative increase in neuronal perikarya size and in neurite outgrowth. This increase in reuptake may have arisen due to increased expression of the dopamine transporter (DAT) in a more complex axonal arbor stimulated by GDNF, although later studies show that GDNF can increase dopamine transport capacity both chronically and acutely (detailed below). As well as transport, the release capacity of dopamine neurons is also increased by up to 380% in midbrain primary cultures in response to potassium chloride (KCl) or latrotoxin stimulation (Pothos et al. 1998). In ventral tegmental area (VTA) cultures GDNF was shown to acutely increase KCl-induced dopamine release twofold and to increase axonal fasciculation (Feng et al. 1999). In a further study it was shown that GDNF produced a threefold increase in autaptic (self-synapsing) currents due to glutamate (Glu) co-release with dopamine and a 100% increase in synaptic terminals after 5–20 days in culture (Bourque & Trudeau 2000).

GDNF is excitatory in midbrain cultures via inhibition of A-type potassium (K+) channels (Yang et al. 2001). This results in a + 7.1 mV shift in the resting membrane potential and a concomitant increase in first spike latency from 132 to 71 ms, an increase in spike frequency from 12.6 to 17.2 Hz, and an increase in membrane conductance. This was seen only in neurons positive for tyrosine hydroxylase (TH) and was dependent upon mitogen-activated protein kinase (MAPK) activation, suggesting it is mediated by GDNF-GFRα-RET activation. GDNF also activates high voltage-gated calcium channels in VTA cultures rapidly and in a reversible manner (Wang et al. 2003) and was confirmed to increase autaptic Glu-based currents, which concurs with previous work (Bourque and Trudeau 2000). It is as yet unknown how these data compare to nigrostriatal preparations as the midbrain preparation is a mixture of VTA and SN cells. Interestingly, it was recently shown that endogenous GDNF upregulation at a threefold level via homozygous 3′ UTR deletion has opposing effects upon dopamine release from the VTA and SN when measured in prefrontal cortex (PFC) and striatum (Str) (Mätlik et al. 2022). 

The mechanism of action for increasing DA production is more well-characterised in SNpc. MAPK and PKA phosphorylate TH at serines 31 and 40, respectively, and increase DA production ~fourfold in rat midbrain cultures in response to acute GDNF (Kobori et al. 2004). This confirms numerous observations of increased DA production and TH phosphorylation in vivo (details below). DA release is also increased ~42% in synaptosomes stimulated with high K+ and in electrically stimulated striatal slices, the effect of which was blocked by antagonising adenosine 2A receptors (A2AR) (Gomes et al. 2006), yet only if GABA receptors were also antagonised. This A2AR-dependent effect was later seen in vivo using an endogenous GDNF overexpression model (Mätlik et al. 2022). The roles of both adenosine and GABA receptors in acute responses to GDNF warrants further investigation.

**Fig. 1:**
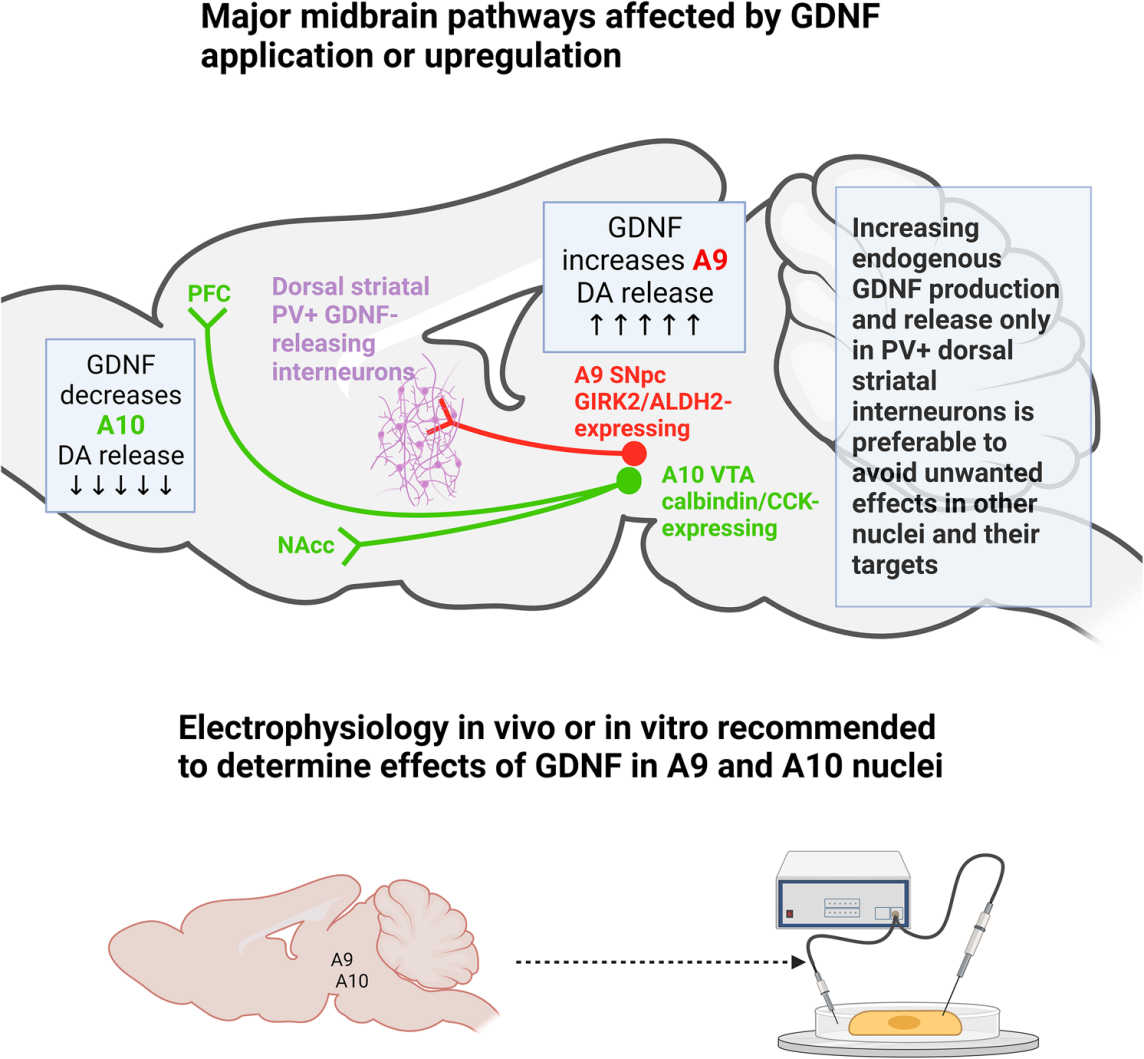
Representation of a mouse brain with major tracts arising from the A9 substantia nigra pars compact (SNpc) and A10 ventral tegmental area. Electrophysiology in acute slices is recommended from both areas as cell types are distinct according to their protein expression profiles, firing properties, projections, and in their response to GDNF. *PV+* parvalbumin-positive GABAergic interneurons; *DA* dopamine; *GIRK2* G-protein-coupled inward-rectifier potassium channel 2; *ALDH2* aldehyde dehydrogenase 2; *CCK* cholecystokinin; *PFC* prefrontal cortex; *NAcc*  nucleus accumbens

Despite evidence of acute increases in DA, in TH phosphorylation, and ERK activation, it was recently shown in midbrain cultures that a long-term increase in GDNF down-regulates all of these activities by decreasing RET, ERK, and AKT phosphorylation by ~50% (Mesa-Infante et al. 2022). Midbrain cultures do however contain a mixture of A9 (SNpc) and A10 (VTA) dopamine neurons that differ markedly in their physiology, morphology, targets, and protein expression profiles (Grealish et al. 2010). A9/SNpc neurons are characterised by expression of G-protein coupled inward-rectifying potassium channel 2 (GIRK2) and ALDH (aldehyde dehydrogenase) and A10/VTA by calbindin and cholecystokinin, with differing firing parameters (Thompson et al. 2005; Lalive et al. 2014). Therefore the effects of GDNF upon A9 and A10 neurons cannot be seen as equivalent, particularly due to differences in their handling of calcium ions and susceptibility to PD (Fu et al. 2016). Further investigation using either A9 cultures or nigrostriatal slices, or A10-derived neurons would be necessary to clarify the acute effects of ectopic GDNF (see Fig. 1). Effects in other dopaminergic nuclei, for example the A8 retrorubral field, remain unexplored (Moaddab & McDannald 2021).

## Exogenous GDNF and Constitutive RET Activation Produce Behavioural Hyperactivity

**Fig. 2:**
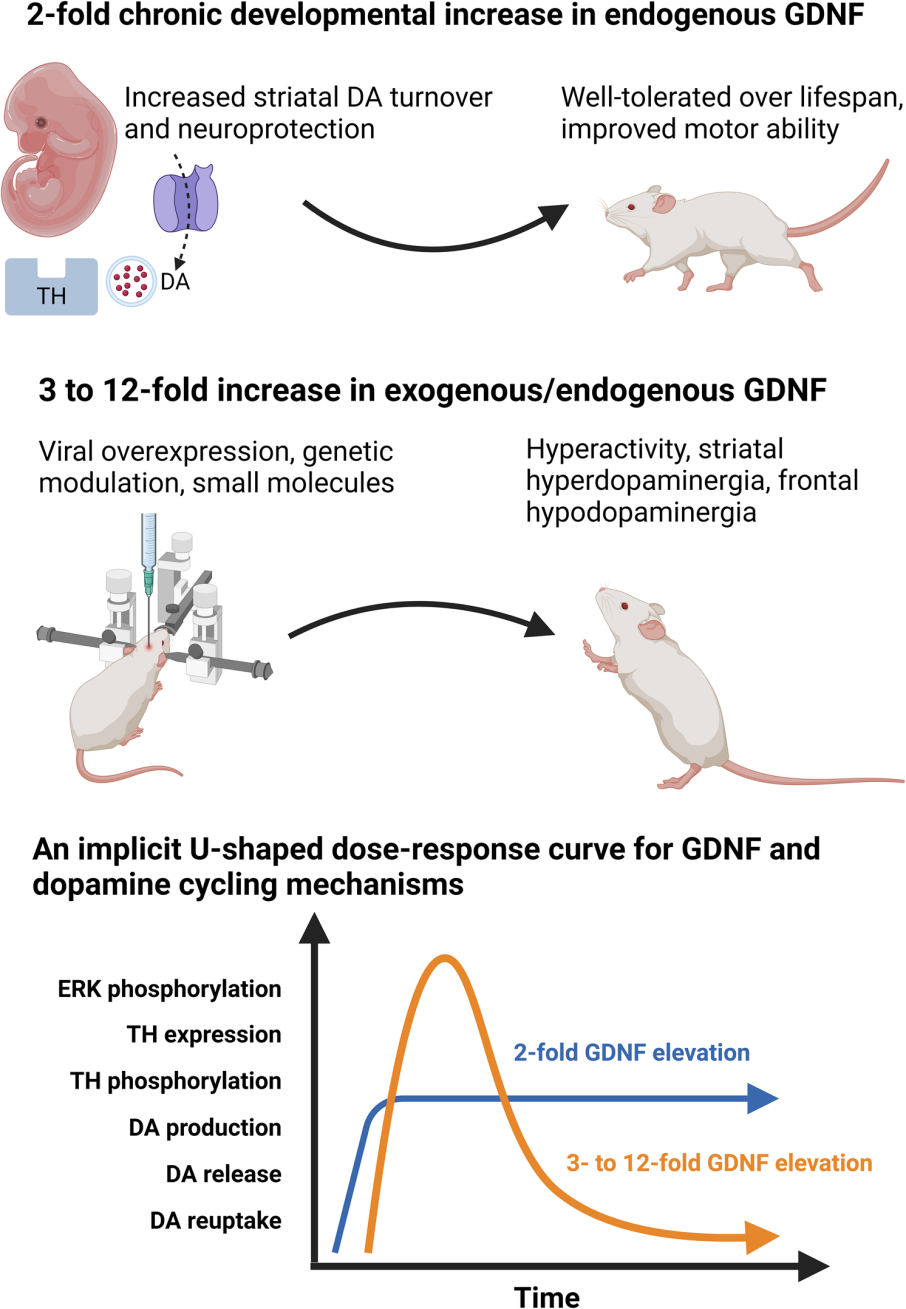
A twofold chronic developmental increase in GDNF expression via genetic upregulation is well-tolerated, increases dopamine cycling and improves motor ability in mice. A three- to 12-fold increase in GDNF via ectopic overexpression or exogenous application produces hyperdopaminergic side-effects and, after an initial increase in dopamine cycling, can lead to a decrease in activity and expression of dopamine and related signalling pathways, suggesting a U-shaped dose–response curve. *ERK* extracellular signal-related kinase; *TH* tyrosine hydroxylase; *DA* dopamine

A single dose of exogenous GDNF directly applied to the nigrostriatal pathway in vivo results in behavioural hyperactivity for several weeks. Initial experiments ejected a single dose of GDNF to the SNpc in young adult rats and saw evidence of hyperactivity lasting 3 weeks (Hudson et al. 1995). This was replicated with injections to both the SNpc and striatum (Martin et al. 1996), in young (Hebert et al. 1996), and in aged rats (Hebert & Gerhardt 1997) and is suggestive of a sustained increase in dopaminergic signalling. GDNF demonstrates a rejuvenating effect in aged rats, who returned to youthful levels of activity and bar-pressing behaviour using implanted GDNF-expressing fibroblasts (Emerich 1996) as well as injections in aged rhesus monkeys which improved hand velocity (Grondin et al. 2003) when striatally injected, as opposed to intracerebroventricularly (Kobayashi et al. 1998). These increases in behavioural activity in response to GDNF are underwritten by locomotor-excited and attenuated bursting of non-locomotor neurons; as seen in aged rats at 24–25 months old using multi-electrode arrays (MEAs) (Stanford et al. 2007). Constitutive activation of the RET receptor also produces marked hyperactivity in a genetic model of multiple endocrine neoplasia type 2B (MEN2B; Mijatovic et al. 2007) with behavioural hypersensitivity to the effects of cocaine. MEN2B mice also show increased sensitivity to amphetamine-induced conditioned place preference (CPP; Kopra et al. 2018), suggesting a hyperdopaminergic phenotype. GDNF and RET signalling therefore appear to have stimulatory effects in youthful animals and produce a more youthful phenotype in aged animals (see Fig. 2 for summary).

## Dopamine Turnover is Increased by Exogenous GDNF or RET Agonist Application In Vivo

Behavioural responses to GDNF injection or increased RET signalling appear to be dopaminergically based as they are blocked by D1 and D2 agonists at low doses (Kobayashi et al. 1998). This has implications for side-effect profiles in potential human treatments for PD as increased dopamine produces “hyperdopaminergic” side-effects, such gambling and other impulse control disorders (Béreau et al. 2018). The above studies generally tested for tissue dopamine levels and/or levels of the dopamine metabolites homovanillic acid (HVA) and/or 3,4-dihydroxyphenylacetic acid (DOPAC); increases of which are indicative of increased dopamine turnover. A three- to fourfold increase in DA turnover in the SN and striatum was indeed seen in the initial experiments (Hudson et al. 1995) and in the striatum (Martin et al. 1996). This increase in turnover was later confirmed via dopamine and metabolite levels measured acutely and chronically in rats after single or multiple doses of GDNF (Hadaczek et al. 2010). Microdialysis in vivo then showed no changes in basal extracellular DA levels yet amphetamine and K+ -stimulated DA release were increased following a single GDNF injection to the SN and increased both HVA and DOPAC, indicating increased DA storage and turnover, respectively (Hebert et al. 1996). This was confirmed in aged rats which also displayed increased K+ and amphetamine-stimulated DA overflow, HVA and DOPAC in the striatum and nucleus accumbens (NAcc, ventral striatum), yet also showed increased extracellular DA (Hebert and Gerhardt 1997); perhaps marking out a more pronounced effect in otherwise healthy yet aged tissue.

More acutely, 24 h post-GDNF injection, microdialysis showed that DA levels are greatly increased in response to methamphetamine yet basal DA levels remained unaffected, despite a ~1.5-fold increase in DA metabolites, again showing increased DA cycling. One week after injection of GDNF, microdialysis showed an increased second DA release in response to 2-pulse K+ stimulation at 70 mM and increased DOPAC and DA—confirmed in tissue post-mortem—further suggesting that exogenous GDNF increases available DA synaptic pools. Similar results were obtained via microdialysis in aged Rhesus monkeys after icv GDNF application, whereby both K+ and amphetamine enhanced striatal DA release and basal DA increased 163% in SN (Grondin et al. 2003). Later studies showed increased DA cycling and better penetration by a variant form of GDNF engineered to have decreased heparin-binding properties due to full glycosylation (Grondin et al. 2019). A twofold increase in DA turnover at day 14 following minimal dosing and better tissue penetration was seen in both rats and rhesus monkeys. The novel RET agonist, BT13, performs similarly to GDNF in acutely increasing both DA release and HVA, indicating increased DA cycling (Mahato et al, 2020).

In aged rats at 24 months striatal injection of GDNF produced increased TH expression and a marked increase in TH phosphorylation at Ser31 in SN (250%) in addition to striatum (40%). ERK1 and ERK2 phosphorylation were increased in SN and striatum, respectively, and microdialysis showed an increase in DA release in response to K+ and amphetamine (Salvatore et al. 2004). Ser31 phosphorylation was also confirmed by Lindgren et al. (2012) in SN via lentiviral overexpression of GDNF. A later study in aged rats at 24–25 months using multi-wire electrode arrays (MEAs) showed that striatal injection of GDNF increased firing of locomotor-associated striatal neurons and attenuated bursting of non-locomotor neurons (Stanford et al. 2007). This points to a mechanism whereby exogenously applied GDNF increases nigro-striatal dopaminergic tone in a sustained manner via increased TH expression and phosphorylation that is dependent upon PKA/ERK signalling. TH phosphorylation was later shown to be increased at Ser19 in SN and both TH and DAT levels were increased in response to striatal GDNF injection (Salvatore et al. 2009). This was accompanied by a decrease in DARPP-32 ipsilaterally and an increase in D1R and DARP-32 phosphorylation contralaterally; suggesting mechanisms of GABAergic regulation in addition to dopamine.

**Fig. 3:**
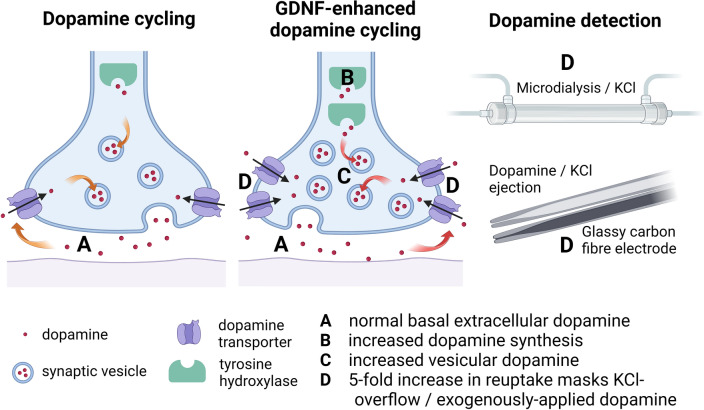
Normal dopamine cycling shown figuratively compared to GDNF-enhanced dopamine cycling. Measurements of extracellular dopamine levels are identical in both scenarios despite increased vesicular dopamine in animals with increased GDNF levels, likely due to a compensatory increase in dopamine reuptake capacity. This affects dopamine detection in overflow experiments via KCl ejection or KCl infusion with microdialysis, or via exogenous dopamine application as any overflow is rapidly taken up presynaptically by DAT. This may lead to masking of dopamine detection (KCl observation is based upon unpublished chronoamperometry data in vivo with glassy carbon fibre electrodes). Blockade of DAT via eg. Nomifensine would allow measurement of overflow. *DAT* dopamine transporter; *KCl* potassium chloride

Of special note is the effect of GDNF upon dopamine reuptake in vivo as the amount of synaptic dopamine is almost exclusively governed by the dopamine transporter (DAT) which fine-tunes behaviour (Giros et al. 1996). T_80_ (time to clear 80% of dopamine) was unchanged following nigral GDNF injection in 4–6 month old rats, yet given a twofold increase in K + -induced DA release, there was calculated to be a 1.75-fold increase in T_C_ (DA clearance) when measured electrochemically in vivo (Hebert et al. 1996). This therefore suggests that DAT activity can compensate for increased DA production, storage, release, and turnover to maintain functional physiological levels of DA in respone to the stimulatory effects of GDNF upon TH levels and activity (see Fig. 3).

## Dopamine Turnover is Increased by GDNF Overexpression or Constitutive RET Activation In Vivo Yet Hypodopaminergia may be Seen at High Levels

Chronic viral ectopic overexpression of GDNF is a robust method that has yielded data on saturating the response to GDNF. Viral overexpression over 6 weeks initially produces similar effects to injection methods; an increase in DA turnover and contralateral amphetamine response was seen, yet after this time DA and metabolite levels returned to normal (Georgievska et al. 2004). Overexpression of GDNF was maintained 12-fold above baseline and led to downregulation of TH that persists, up to 24 weeks post-viral injection in this study and for 13 months in a previous study, potentially indicating a saturating response to GDNF (Rosenblad et al. 2003). The latter study showed no effect upon D1 or D2 receptor density, suggesting compensatory mechanisms. Similarly, an AAV model of delivery over 5 weeks in rats again produced a 12-fold increase in GDNF expression and led to a decrease in TH and TH phosphorylation at Ser40, a decrease in dopamine and in reuptake; although a more modest threefold increase in GDNF had no effect on TH levels. Overexpression via the *Gfap* promotor in glial cells at three to tenfold levels compared to wild-type litter-mates produced a similar hypodopaminergic phenotype with reduced DA, HVA, and K+ -induced DA efflux (Sotoyama et al. 2017).

A more refined attempt at overexpression employed a (tTA)/tTA-responsive promotor system under CAMKIIa to overexpress GDNF at two- to threefold levels in cortex, hippocampus and brainstem in any cells that normally express CAMKIIa (Kholodilov et al. 2004). These mice displayed an increased response to amphetamine yet normal DA levels, normal electrically evoked DA release in striatal slices and normal striatal innvervation, normal striatal TH and DA synptic bouton levels, yet more dense DAergic innnvervation in the prefrontal cortex (PFC). This suggets a much greater effect upon A10 neurons than A9 in this model and is therefore of limited utility in striatum.

Further investigation of mice with constitutively active RET signalling (MEN2B) showed increased TH activity and DA synthesis yet, unusually, no increase in K+ -stimulated DA release via microdialysis (Mijatovic et al. 2008). Taking a finer approach with in vivo voltammetry showed an increase in DA release via medial forebrain bundle (MFB) electrical stimulation and increased DA uptake, concurring with the effects seen in chronic GDNF injection shown previously in rats (see Hebert et al. 1996). where increased storage and release capacity of DA were established.

Later work has shown that GDNF is synthesised primarily in parvalbumin-positive interneurons of the striatum (Hidalgo-Figueroa et al. 2012). Direct targeting of these cellular sources of GDNF in the striatum may be possible via specific combinations of ligands (Enterría-Morales et al. 2020) or genetically (Kumar et al. 2015; Mätlik et al. 2022). The latter two papers showed endogenous upregulation of GDNF through heterozygous blanking of the via 3′ UTR of the *Gdnf* gene. A twofold increase in GDNF expression in these natively GDNF-expressing cells led to a concomitant increase in dopamine cycling that was reflected in a fivefold increase in dopamine reuptake capability, without observable side-effects (Kumar et al. 2015).

## Behavioural Responses to Endogenous GDNF Upregulation In Vivo are Dose-Dependent

Endogenous overexpression of GDNF, ie. only in those cells that would natively express GDNF, presents a novel approach to augmenting and protecting the dopamine system prior to degeneration. Removing binding sites in the 3′ untranslated region (UTR) of the mouse *Gdnf* gene itself prohibits the binding of regulatory microRNAs and produces an increase in both GDNF mRNA and protein. The effect is strong enough that a heterozygous animal produces a twofold increase in GDNF (Kumar et al. 2015) without an increase in basal extracellular DA, yet a 40% increase in DOPAC and a 40% increase in DA release in slices following a single electrical stimulus, indicating enhanced storage and dopamine cycling. This is further confirmed by a fivefold increase in maximal uptake rate, suggesting enhanced DAT function to compensate for a greater DA release pool. Such marked changes in DA signalling had only minimal effects upon behaviour as these animals displayed no hyperactivity or indeed any behavioural phenotype, yet had enhanced motor function including balance and grip strength (Mätlik et al. 2018). In aged animals at 17–19 months the effect was persistent; no changes in behaviour such as hyperactivity or anxiety were observed and a more juvenile-like state of enhanced grip strength, motor learning, and vertical grid ability were seen with a minor increase in TH+ cells in SNpc (Turconi et al. 2020). This was further confirmed via a separate technique using antisense long non-coding RNAs that promote transcription of sense mRNAs (SINEUP; containing a SINEB2 sequence to up-regulate transcription) where GDNF protein and synaptic dopamine were increased in a twofold manner (Espinoza et al. 2020). Similarly, DA release in response to stimulation in vitro was increased, as was total tissue dopamine, yet without hyperactivity. This would suggest that off-target effects of GDNF, perhaps via NCAM and Syndecan-3 may be responsible for side-effect profiles following exogenous GDNF application. Therefore, endogenous upregulation appears to be the best-tolerated and the most target-specific method for increasing GDNF signalling.

In further support of the endogenous elevation route, the *Gdnf* 3′UTR has been excised via Cre-LoxP. Homozygous removal of the 3′UTR resulted in a more than threefold increase in *Gdnf* mRNA and affected prepulse inhibition in mice, suggesting schizophrenia-like behaviour (Mätlik et al. 2022), in contrast to heterozygous blanking (Kumar et al. 2015), and that striatal dopamine reuptake was greatly increased in vitro, reflecting findings in vivo. Tissue DA was increased in striatum yet greatly decreased in VTA and prefrontal cortex (Mätlik et al. 2022). Increased water intake and visits to the water were also seen, perhaps indicating a lack of behavioural control or an element of compulsivity. This highlights both the very different nature of the A9 and A10 dopaminergic nuclei and the importance of finding an appropriate level of GDNF overexpression due to potential cognitive and behavioural effects.

One important methodological consideration arose in measuring extracellular dopamine levels, as reported in Kumar et al. (2015). Electrochemical recordings to assess dopamine reuptake rate showed a fivefold increase in reuptake in GNDF overexpressing mice versus wild-type animals. This increase in reuptake was able to mask extracellular dopamine; for a given quantity of dopamine ejected, a markedly lower level of extracellular dopamine was seen in GDNF transgenic animals that completely masked extracellular dopamine peaks at physiological concentrations. Blockade of the dopamine transporter (DAT) combined with electrical or chemical stimulation of DA release can better provide information on DA overflow in response to GDNF.

## Outside GDNF-GFRa1-RET

There is evidence for GDNF signalling via multiple pathways and for unexplored roles in maintaining normal striatal function outside of canonical RET signalling. NCAM1, integrins αv and β1, and N-cadherin induce neurite outgrowth, proliferation, survival, and influence axon guidance (Chao et al. 2003; Paratcha et al. 2003; Cao et al. 2008; Zuo et al. 2013; Ibáñez et al. 2020) and several of these adhesion proteins may act as receptors for GDNF bound to its cognate receptor, GFRα1. Matrix-bound GDNF may also signal via Syndecan-3 (Bespalov et al. 2011) which in turn affects GABAergic neuronal migration and is neither RET- nor NCAM-dependent (Pozas & Ibanez 2005; Canty et al. 2009; Marshall et al. 2021). Although RET and GFRα1 are expressed throughout development and adulthood in midbrain dopamine neurons, GFRα1 is expressed in the absense of RET in striatal neurons (Kramer & Liss 2015). Additionally, GDNF does not appear to be essential for the propagation and maintenance of dopamine neurons in vivo (Jain et al. 2006; Kopra et al. 2015) yet it is necesssary for their long-term maintenance and function in concert with other growth factors (Conway et al. 2020; Li et al. 2022).

## Conclusion and Open Questions

A clearer framework for working with GDNF in terms of overexpression, localisation, and off-target effects is now evident. This highlights the pitfalls of exogenous application and the benefits of endogenous upregulation as well as the marked effects of GDNF upon DA transport. DA overflow in systems with greatly increased dopamine transporter activity may not be an accurate marker of DA release as accurate measurement may be masked by increased reuptake kinetics (see Fig. 3). This is due to the remarkable self-righting capacity of the nigrostriatal DA system, that appears to falter only under very significant neurodegeneration as seen in the late stages of Parkinson’s disease (Cheng et al. 2010). GDNF mimetics and RET agonists appear promising - provided that they can be targeted and dosed appropriatdly - yet the targeting of endogenous sources of GDNF in the striatum appears to be the most desirable target. 95% of striatal GDNF is produced in the parvalbumin-positive interneurons that together form a fast-spiking, electrotonically connected network that provides trophic support to dopamine terminals (Koós & Tepper 1999; Tepper et al. 2010; d’Anglemont de Tassigny et al. 2015; Enterría-Morales et al. 2020). Genetic modulation of GDNF expression in these interneurons or the development of specific ligands are important focal points for future research to protect, support, and re-grow the dopamine neurons lost during the progression of Parkinson’s disease. GABAergic or purinergic systems should also be investigated as potential adjunct therapies. Differentiating between the effects of GDNF in discrete nuclei is of key importance as there appear to be opposing effects in A9 versus A10 dopaminergic neurons whereby GDNF is either stimulatory or inhibitory for dopamine regulation**,** respectively. This may affect the tolerability of future therapies in humans due to effects upon cognitive and behavioural functions. 

## Data Availability

Enquiries about data availability should be directed to the authors.
